# Quantifying the effect of air pollution and temperature on hospitalization costs for chronic lower respiratory diseases

**DOI:** 10.3389/fpubh.2025.1724510

**Published:** 2025-12-04

**Authors:** Huanhuan Jia, Shuqi Xu, Shang Gao, Liangwen Ning, Xihe Yu

**Affiliations:** 1 School of Management, Xuzhou Medical University, Xuzhou, Jiangsu, China; 2 School of Public Health, Jilin University, Changchun, Jilin, China

**Keywords:** hospitalization costs, chronic lower respiratory diseases, air pollution, temperature, inpatient

## Abstract

**Background:**

Environmental exposure is a crucial factor contributing to the increasing health and economic burden of chronic lower respiratory diseases (CLRDs). This study aims to quantify the effects of environmental exposure on hospitalization costs associated with CLRDs and their population heterogeneity, thus providing a scientific basis for formulating more precise and effective cost-control policies.

**Methods:**

Hospital admission records from seven major tertiary hospitals in Changchun from 2017 to 2020 were collected, as were environmental monitoring and meteorological data. A generalized additive model and distributed lag nonlinear model were employed to identify the exposure–response relationships and lag effects of pollutants and temperature.

**Results:**

A total of 23,569 patients with CLRDs were included. Most of the participants were male (50.83%) and aged 60 years and older (63.42%), with hospitalization costs accounting for 54.22 and 67.96% of total costs, respectively. When the concentrations of PM2.5, PM10, SO_2_, or NO_2_ increased by 10 μg/m^3^, the hospitalization costs on the same day increased by 1.78, 0.87, 8.30, and 4.43%, respectively. The cumulative lag effects of PM2.5, SO_2_, and NO_2_ peaked after 6 days, whereas those of PM10 peaked after 5 days. Inhalable particulate matter had a greater effect on children aged 0–14 years and those aged 60 years and older, whereas SO_2_ and NO_2_ had greater effects on those aged 15–59 years. With respect to temperature, the hospitalization costs of people aged 60 and above increased by 8.70% on low-temperature days, and the exposure response ratio from lag day 0 to lag day 5 showed a statistically significant gradual decrease. Additionally, on lag days 5 and 11, the hospitalization costs of female patients with CLRDs increased by 3.35 and 3.92%, respectively.

**Conclusion:**

A significant association was observed between increased air pollutant concentrations and the hospitalization costs of patients with CLRDs, with heterogeneity among different populations. Specifically, the effects of SO_2_ and NO_2_ were more pronounced, particularly in the 15–59 age group. Moreover, an increase in particulate matter concentration had a greater effect on children and older population. Extreme low-temperature weather significantly affected the hospitalization costs of older population and women with CLRDs.

## 1 Introduction

Data from the World Health Organization (WHO) indicated that in 2019, direct medical costs attributable to chronic obstructive pulmonary disease (COPD) and chronic bronchitis worldwide exceeded $38 billion, with low-income countries experiencing a cost growth rate 2.3 times greater than that of developed regions (1). Research has indicated that the increased prevalence and health care burden of respiratory diseases are, to a significant extent, associated with environmental exposure (2, 3). A substantial body of epidemiological research has confirmed the significant and complex effects of air pollution (PM2.5, SO_2_, NO_2_, etc.) and temperature fluctuations on respiratory health. Exposure to particulate matter in the air can easily trigger inflammatory damage, oxidative stress responses, and a variety of other biological effects (4). Short-term exposure to high concentrations of PM2.5 significantly increases the number of visits for patients with emphysema and COPD (5). Additionally, exposure to SO_2_, NO_2_, O_3_, and CO has been proven to be closely related to the incidence and mortality of respiratory diseases (6–8). Furthermore, there are diverse patterns of exposure–response relationships between temperature and residents’ health, such as U-shaped, V-shaped, or J-shaped patterns, which reflect significant differences in health risks corresponding to different temperature values. Both high and low temperatures can increase the risk of respiratory diseases and other health issues (9–11). Notably, the interactive effects of pollutants and climate can produce synergistic amplification effects, with winter air pollutants having a far greater effect on respiratory diseases than air pollutants in other seasons do (12). Moreover, older population and children are more susceptible (13, 14).

Air pollutants and extreme temperatures not only contribute to the increase in disease incidence rates, but they also directly drive the consumption of medical resources and sharply increase health care costs (6, 15, 16). From 2008 to 2018, health care expenditures for COPD, a predominant noncommunicable disease in the European Union, constituted 1.7% of total health care spending, with significant underreporting (17, 18). In 2016, the United States recorded $17 billion in medical expenditures for respiratory diseases (19). In Canada, the direct economic cost for each asthma patient ranges between $366 and $647, with indirect costs—including labor force loss, vitality decline, and functional impairment—being even more substantial (20). Italian studies have revealed that health care expenditures for COPD patients account for 6% of total expenditures, imposing a considerable economic burden (21). In addition, research conducted in Tehran, Iran, suggested that reducing the PM2.5 concentration to 2.4 μg/m^3^ could yield an estimated annual economic benefit of $1.47 billion for patients with COPD (22). Similarly, in China, respiratory diseases consume a significant portion of health care resources, with treatment costs representing a high proportion at both the national and local levels (23, 24). This phenomenon is particularly pronounced in industrialized and climate-sensitive areas. Taking Jilin Province in China as an example, the region’s history as an old industrial base and its location in the cold climate zone at 43°N latitude have led to a unique vicious cycle of “chronic exposure–lung function damage–medical cost accumulation” (25). However, a significant knowledge gap remains in environmental health economics research: How do air pollutants and temperature parameters differentially affect the health economic burden of chronic lower respiratory diseases (CLRDs)? Is there population heterogeneity in their mechanisms of action?

Therefore, current economic research on CLRDs has three major limitations. First, the effect of the environment on treatment costs is often indirectly derived through incidence rates and other intermediary effects, and a direct quantitative analysis of the effects of air pollutants and temperature on treatment costs is lacking. Second, there are inherent differences in pathological mechanisms, treatment cycles, and cost response patterns among different types of respiratory system diseases, but the literature lacks a systematic analysis of the associations between environmental exposure (air pollutants and temperature) and chronic respiratory system diseases. Finally, empirical research on old industrial bases and cold climate areas remains scarce. These areas may exhibit unique cost-driving mechanisms because of the combined effects of historical pollution and extreme temperatures, but this mechanism has not been fully revealed or understood.

Therefore, in this study, Jilin Province, with its typical temperature characteristics and environmental features, was selected as a case study area. By integrating time series data on treatment costs and environmental monitoring data, this study aims to quantify the specific effects and differences in the effects of major air pollutants and temperature on the hospitalization costs of CLRDs. This study further identifies vulnerable populations, providing a scientific basis and decision support for cold industrial cities to develop more precise and effective cost control policies.

## 2 Methodology and data

### 2.1 Data collection

#### 2.1.1 Case information

In accordance with the International Classification of Diseases 10th Revision (ICD-10) codes J10-J18, which define CRLDs, this study compiled admission records from seven major tertiary hospitals in Changchun city from January 1, 2017, to December 31, 2020. The collected case data comprised patient demographics, including age and sex, dates of admission and discharge, diagnostic outcomes, and hospitalization costs. The “hospitalization costs” variable represents the direct medical expenses billed by the hospital for the entire inpatient episode. It encompasses costs related to medications, laboratory tests, imaging examinations, therapeutic procedures, and nursing services.

#### 2.1.2 Meteorological and air pollutant information

To explore the potential effects of meteorological conditions on the incidence of influenza and pneumonia, this study extracted the daily average temperature (°C) data of Changchun city from the National Meteorological Science Data Center^1^ from January 1, 2017, to December 31, 2020. In addition, to ensure the accuracy of the analysis results and eliminate interference from other potential meteorological factors, we simultaneously collected average air pressure (hPa) and average relative humidity (%) data during the same period. The monitoring data of the main air pollutants in Changchun city, Jilin Province, from January 1, 2017, to December 31, 2020, were collected from the China Air Quality Online Monitoring and Analysis Platform, including the concentration data of six main air pollutants, namely, PM2.5, PM10, SO_2_, NO_2_, O_3_, and CO. O_3_ is reported as the daily maximum 8-h average concentration, and PM2.5, PM10, SO_2_, NO_2_, and CO are reported as the 24-h average concentrations.

To more precisely understand the uniqueness of Changchun’s air quality and its position nationwide, we referred to the “Bulletin of China’s Ecological and Environmental Status” issued by the Ministry of Ecology and Environment of the People’s Republic of China from 2017 to 2020. By comparing the national average temperature and the average level of air pollutant concentrations, the unique temperature characteristics of Changchun were determined, and the primary air pollutants in this area were identified, laying a foundation for subsequent in-depth analysis.

As shown in Table 1, from 2017 to 2020, the average temperature in Changchun was 7.20 °C, which was significantly lower than the national average temperature. The concentrations of the six major air pollutants assessed in this study tended to decrease from 2017 to 2020. When the Changchun values were compared with the national average, the average concentrations of PM2.5 and PM10 in Changchun in 2017, 2019, and 2020 were greater than the national average values. The average concentration of PM2.5 in Changchun in 2020 was 1.27 times the national average. The average concentration of SO_2_ in Changchun in 2017 and 2018 was higher than the national average, and the average concentration of SO_2_ in Changchun in 2017 was 1.47 times the national average. The average concentration of NO_2_ in Changchun from 2017 to 2020 was higher than the national average, and the average concentration of NO_2_ in Changchun in 2020 was 1.32 times greater than the national average. However, the average concentrations of CO and O_3_ in Changchun from 2017 to 2020 were lower than the national average. Thus, compared with the country as a whole, Changchun was characterized by lower temperatures and higher concentrations of the key air pollutants PM2.5, PM10, SO_2_, and NO_2_. Therefore, this study evaluated PM2.5, PM10, SO_2_, and NO_2_ as the primary pollutants in Changchun for subsequent analysis.

**Table 1:** Daily average temperature and air pollutant concentrations in Changchun and China, 2017–2020.

Variables	2017		2018		2019		2020	
	Changchun	China	Changchun	China	Changchun	China	Changchun	China
Temperature (°C)	7.05	10.39	6.92	10.09	7.65	10.34	7.18	10.25
PM_2.5_ (μg/m^3^)	46.05	43	32.23	39	37.85	36	42.06	33
PM_10_ (μg/m^3^)	81.30	75	61.19	71	65.76	63	61.35	56
SO_2_ (μg/m^3^)	26.38	18	14.63	14	10.92	11	9.90	10
NO_2_ (μg/m^3^)	40.17	31	32.43	29	33.60	27	31.73	24
CO (mg/m^3^)	1.18	1.7	0.77	1.5	0.74	1.4	0.71	1.3
O_3_ (μg/m^3^)	90.13	149	75.22	151	80.38	148	79.99	138

### 2.2 Statistical analysis

This study adopted a time series design to investigate the associations among short-term exposure to air pollutants, temperature, and hospitalization costs for CLRDs.

First, according to the consumer price index (CPI) published in the “Statistical Yearbook” released by the Jilin Provincial Bureau of Statistics, the treatment costs of respiratory diseases from 2017 to 2020 were adjusted by deflation to ensure the comparability of the data. Descriptive analysis methods were subsequently used to analyze the patients’ demographic characteristics and treatment costs. According to the age categorization of China’s previous censuses, individuals were divided into three age groups: children aged 0–14 years, individuals aged 15–59 years, and people aged 60 years and older.

The generalized additive model (GAM), proposed by Hastie and Tibshirani in 1990, expands the concept of the generalized linear model (GLM) by fitting the independent variables in a nonparametric function form to estimate the relationships between the independent variables and the dependent variables. GAMs have been widely used in environmental epidemiology to explore the relationship between air pollutant exposure and the incidence or mortality of diseases (25, 26).

In this study, a GAM was used to analyze the association effect between the change in air pollutant concentration and the hospitalization cost of CLRDs. Specifically, this paper analyzes the changes in hospitalization costs associated with CLRDs on the current day (lag0) and the N-day lag period (lag1 - N) when the air pollutant concentration increases by 10 μg/m^3^, that is, the excess risk (ER). In previous epidemiological studies analyzing the association between changes in the external environment and changes in population health, the selection of lag days N was often determined according to the research purpose, and there were large differences (27–29). An analysis revealed that a shorter lag period easily masks the lag effect of the environment, whereas a longer lag period is prone to dilute the significance of the association between the environment and hospitalization costs. Moreover, practical significance is absent with a longer lag period, resulting in an unstable model fitting effect. Therefore, in conjunction with previous studies on the lag response relationship of air pollutant exposure, the lag period N in this study was determined to be 14 days.

Moreover, because hospitalization for CLRDs is a low-probability event and its distribution is an approximately Poisson distribution (30, 31), this study established a GAM based on a quasi-Poisson distribution. In this model, hospitalization costs are regarded as the outcome, and the daily average pollutant concentration is regarded as the predictor. To account for potential temporal confounders, the model incorporated a smooth function of calendar time to control for long-term trends and seasonality. Furthermore, previous studies have indicated that working days and holidays affect the number of hospital admissions for diseases (32). Therefore, the day of the week (DOW) was included as a categorical variable to control for weekly patterns in hospital admissions. Public holidays were also controlled for using a binary variable (Holiday). Moreover, the model smoothed the meteorological factors.

The expression of the GAM is as follows:


$$\begin{array}{l} \log(\mathrm{Yi})=\beta (\mathrm{Ci})+s(\text{Time},\mathrm{df})+s(\mathrm{MT},\mathrm{df})+s(\mathrm{RH},\mathrm{df})\\ +s(\mathrm{MB},\mathrm{df})+\mathrm{DOW}+\text{Holiday}+\alpha \end{array}$$

In the equation, *Yi* is the expected hospitalization cost of the CLRDs on the *ith* day aggregated daily. *Ci* is the average concentration of air pollutants. *β* is the regression coefficient estimated by the model. *Time* is the date variable. To eliminate the influence of meteorological factors on the results, the average temperature, average relative humidity, and average relative air pressure are included in the model and are denoted as *MT*, *RH*, and *MB*, respectively. *ns* represents the natural smooth spline function. *df* refers to the degrees of freedom. The degrees of freedom of the average temperature, average relative humidity, and average relative air pressure are set to 3 on the basis of previous studies, whereas the degrees of freedom of time are determined according to the Akaike information criterion (AIC) (33). *α* is the intercept. Finally, the different degrees of freedom of time are controlled, a two-pollutant model is constructed to eliminate the influence of other pollutants, and a sensitivity analysis is conducted to verify the robustness of the model (34, 35).

The distributed lag model (DLM) has been widely used in studies of the health effects of meteorological factors. However, this model assumes a linear exposure–response relationship, whereas in reality, many exposure–response relationships (such as temperature–mortality) exhibit nonlinear relationships, such as U and V, in which cases the distributed lag model is not fully applicable (36). To address this issue, in 2006, Armstrong first proposed the distributed lag nonlinear model (DLNM) and applied it in epidemiology (37). The advantage of the DLNM is that it can simultaneously consider the lag effect of exposure factors and the nonlinear exposure–response relationship. In this study, a cross-basis matrix was constructed for the hospitalization costs of patients with CLRDs and temperature data. The hospitalization cost was set as the dependent variable, and the quasi-Poisson link function was used for fitting. To control for the seasonal, long-term trend, and day-of-the-week effects, the DLNM was used to fit the association between temperature and the hospitalization cost of CLRDs. Specifically, we used a natural cubic spline function of time to model long-term and seasonal trends and included ‘DOW’ and ‘Holiday’ as categorical variables. Moreover, the natural cubic spline function was selected for the two-dimensional cross-basis matrix, with the average temperature as the reference level. By comparison with the reference level, the associations between high temperatures (P2.5 percentile of the temperature sequence) and low temperatures (P97.5 percentile of the temperature sequence) at different lag periods and the change in the hospitalization cost of CLRDs, namely, RR and ER, were calculated. Additionally, similar to the GAM, the maximum lag period was set to 14 days.

The expression of the DLNM is as follows:


$$\begin{array}{l} \log(\mathrm{Ei})=\beta \;\text{basis}.\text{temp}+\mathrm{ns}(\text{Time},\mathrm{df})+\mathrm{ns}(\mathrm{RH},\mathrm{df})\\ +\mathrm{ns}(\mathrm{MB},\mathrm{df})+\sum \mathrm{ns}(\mathrm{Pl},\mathrm{df})+\mathrm{DOW}+\text{Holiday}+\alpha \end{array}$$

In the equation, *base, temp* is the cross-basis matrix. To eliminate the influence of air pollutants and other meteorological factors, the pollutants, average relative humidity, and average relative air pressure are included in the model and are denoted as *pl*, *RH*, and *MB*, respectively. The meanings of the other variables are the same as those in the GAM. Finally, by controlling the different degrees of freedom of time, a sensitivity analysis was performed to verify the robustness of the model.

## 3 Results

### 3.1 Demographic characteristics and costs of patients with CLRDs

From 2017 to 2020, a total of 23,569 patients were admitted for CLRDs at the investigated hospitals, with a total hospitalization expense of 404.27 million yuan. Cases involving men were more common (50.83%), with a cost proportion of 54.22%. The majority of cases occurred in the population aged 60 years and older (63.44%), and the corresponding cost proportion was 67.96%. Moreover, although the proportion of nonsurgical patients was 88.87%, the surgical patients (11.43% of the population) accounted for 25.21% of the treatment costs (Table 2).

**Table 2:** Demographic characteristics and costs of patients with CLRDs.

Variables	Number		Expense	
	*N*	%	Yuan (Million)	%
Gender				
Male	11,980	50.83	219.18	54.22
Female	11,589	49.17	185.09	45.78
Age				
0–14	2,320	9.84	13.83	3.42
15–59	6,301	26.73	115.71	28.62
Age 60 and above	14,948	63.42	274.72	67.96
Surgery				
No	20,875	88.57	302.36	74.79
Yes	2,694	11.43	101.91	25.21
Total	23,569	100.00	404.27	100.00

### 3.2 Association effect between air pollutants and CLRD hospitalization expenses

**Figure 1:**
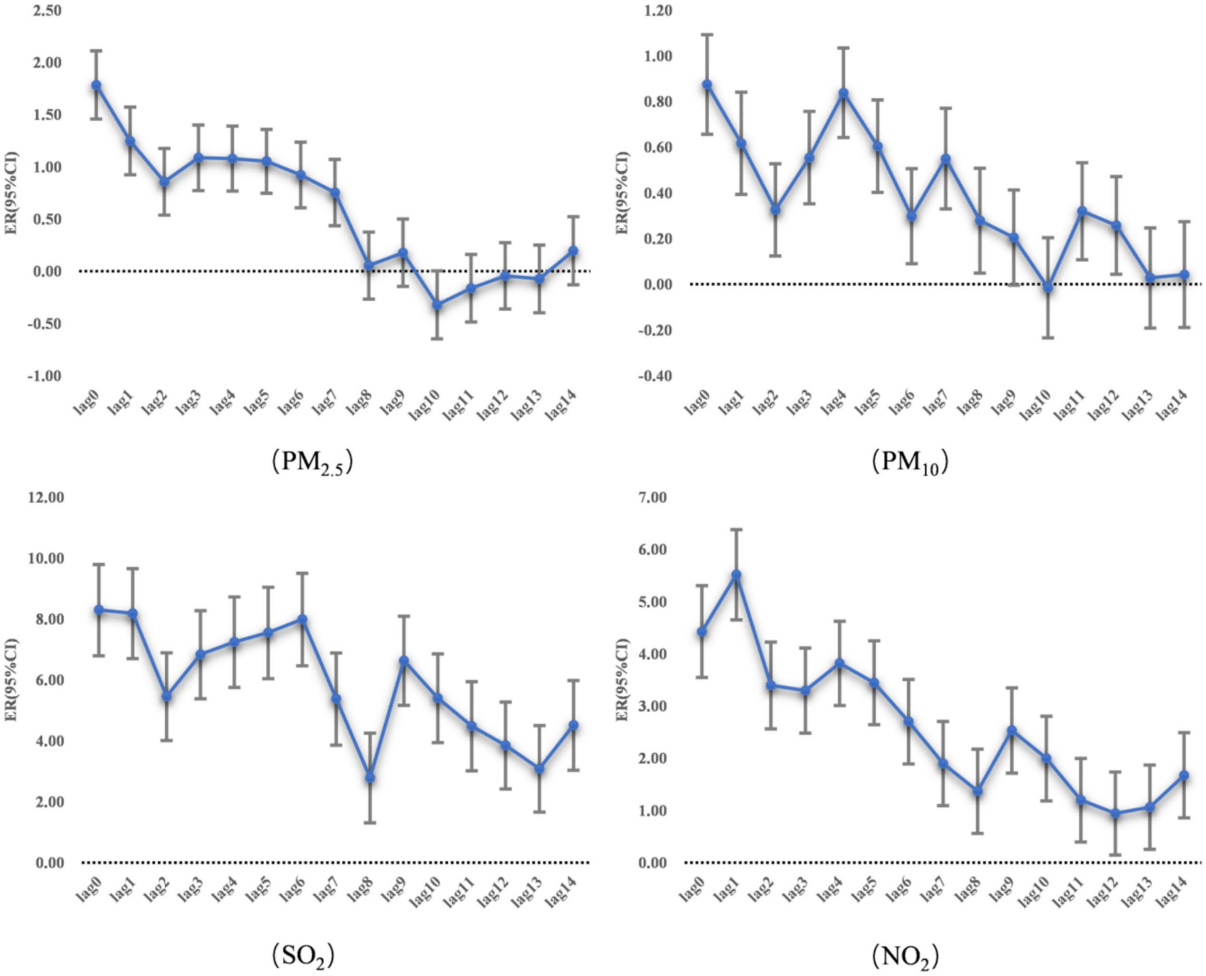
Association of changes in air pollutant concentration with CLRD hospitalization costs.

When the concentration of PM2.5 increased by 10 μg/m^3^, the hospitalization expenses of CLRDs on the same day increased by 1.78% (95% CI: 1.46, 2.11%), and the ER decreased from lag day 0 to lag day 14. When the concentration of PM10 increased by 10 μg/m^3^, the hospitalization expenses on the same day increased by 0.87% (95% CI: 0.66, 1.09%), and the ER decreased from lag day 0 to lag day 14. When the concentration of SO₂ increased by 10 μg/m^3^, the hospitalization expenses associated with CLRDs on the same day increased by 8.30% (95% CI: 6.81, 9.81%). The ER from lag day 0 to lag day 14 first decreased but then increased, with the lowest ER on lag day 8, and the hospitalization expenses increased by 2.79% (95% CI: 1.33, 4.27%). When the concentration of NO₂ increased by 10 μg/m^3^, the CLRD hospitalization expenses on the same day increased by 4.43% (95% CI: 3.55, 5.31%), the ER on lag day 1 was the largest, and the hospitalization expenses increased by 5.52% (95% CI: 4.66, 6.38%). The ER from lag day 0 to lag day 14 exhibited a fluctuating decreasing trend, with the lowest ER occurring on lag day 12, and the hospitalization expenses increased by 0.94% (95% CI: 0.15, 1.74%). The detailed data are presented in Table 3 and Figure 1.

**Table 3:** Single-day lag effects of a 10 μg/m^3^ increase in air pollutant concentration on CLRD hospitalization costs.

Lay days	PM_2.5_	PM_10_	SO_2_	NO_2_
lag0	**1.78 (1.46, 2.11)**	**0.87 (0.66, 1.09)**	**8.30 (6.81, 9.81)**	**4.43 (3.55, 5.31)**
lag1	**1.25 (0.92, 1.57)**	**0.62 (0.39, 0.84)**	**8.18 (6.72, 9.67)**	**5.52 (4.66, 6.38)**
lag2	**0.86 (0.54, 1.18)**	**0.33 (0.12, 0.53)**	**5.46 (4.03, 6.91)**	**3.40 (2.57, 4.23)**
lag3	**1.09 (0.77, 1.40)**	**0.55 (0.35, 0.76)**	**6.84 (5.40, 8.29)**	**3.30 (2.49, 4.12)**
lag4	**1.08 (0.77, 1.39)**	**0.84 (0.64, 1.03)**	**7.25 (5.77, 8.74)**	**3.82 (3.01, 4.63)**
lag5	**1.05 (0.75, 1.36)**	**0.60 (0.40, 0.81)**	**7.55 (6.06, 9.06)**	**3.45 (2.65, 4.25)**
lag6	**0.92 (0.61, 1.24)**	**0.30 (0.09, 0.51)**	**7.99 (6.48, 9.52)**	**2.70 (1.90, 3.51)**
lag7	**0.75 (0.44, 1.07)**	**0.55 (0.33, 0.77)**	**5.38 (3.88, 6.91)**	**1.90 (1.10, 2.71)**
lag8	0.05 (−0.27, 0.38)	**0.28 (0.05, 0.51)**	**2.79 (1.33, 4.27)**	**1.37 (0.57, 2.18)**
lag9	0.18 (−0.15, 0.50)	**0.20 (0.00, 0.41)**	**6.64 (5.18, 8.11)**	**2.53 (1.72, 3.35)**
lag10	−0.32 (−0.65, 0.00)	−0.02 (−0.23, 0.2)	**5.41 (3.96, 6.87)**	**2.00 (1.19, 2.81)**
lag11	−0.16 (−0.49, 0.16)	**0.32 (0.11, 0.53)**	**4.49 (3.03, 5.96)**	**1.20 (0.40, 2.00)**
lag12	−0.04 (−0.36, 0.27)	**0.26 (0.04, 0.47)**	**3.85 (2.44, 5.29)**	**0.94 (0.15, 1.74)**
lag13	−0.07 (−0.4, 0.25)	0.03 (−0.19, 0.25)	**3.09 (1.68, 4.52)**	**1.07 (0.26, 1.88)**
lag14	0.20 (−0.13, 0.52)	0.04 (−0.19, 0.27)	**4.52 (3.05, 6.00)**	**1.68 (0.86, 2.50)**

ER, excess risk; CI, confidence interval. [ER% (95% CI)]. Bold values indicate a statistically significant difference.

**Figure 2:**
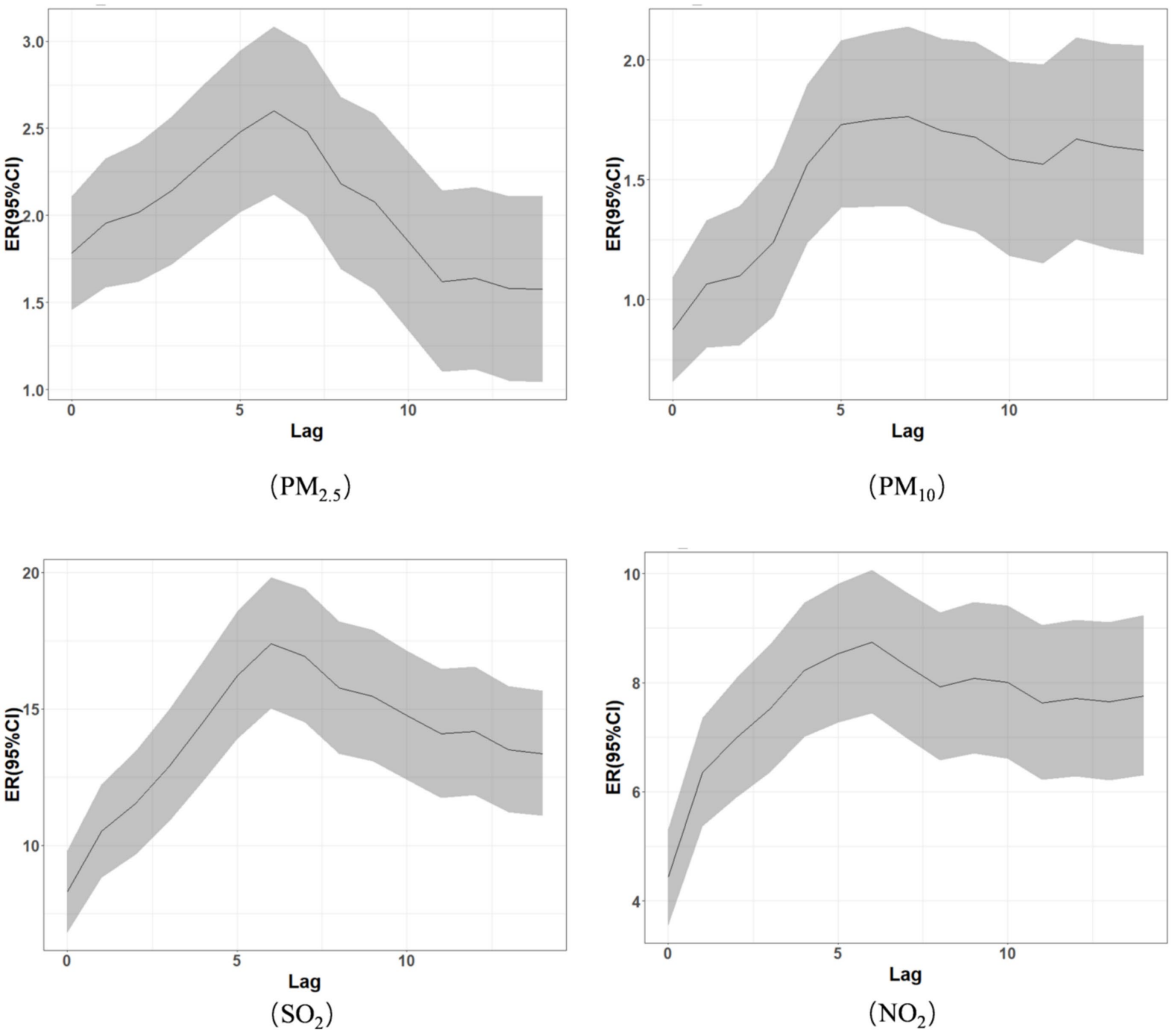
Cumulative lag effect of air pollutant concentration changes on the CLRD hospitalization costs.

The results of the cumulative lag effect demonstrate that when the concentrations of PM2.5, PM10, SO₂, and NO₂ increased by 10 μg/m^3^, the cumulative lag effect of CLRD hospitalization expenses first tended to increase but then decreased. The cumulative lag effect after the increase in the concentrations of PM2.5, SO₂, and NO₂ reached a maximum at lag days 0–6, and the hospitalization expenses increased by 2.60% (95% CI: 2.12, 3.09%), 17.40% (95% CI: 15.01, 19.83%), and 8.75% (95% CI: 7.44, 10.07%), respectively. The cumulative lag effect after the increase in the concentration of PM10 reached a maximum at lag days 0–5, and hospitalization expenses increased by 1.73% (95% CI: 1.38, 2.08%). See Table 4 and Figure 2 for details.

**Table 4:** Cumulative lag effect of air pollutant concentration changes on CLRD hospitalization costs.

Air pollutant	Cumulative lag effect in 0–14 days	Maximum cumulative lag effect	
		Lag day	Cumulative lag effect
PM_2.5_	**1.58 (1.04, 2.11)**	6	**2.60 (2.12, 3.09)**
PM_10_	**1.62 (1.19, 2.06)**	5	**1.73 (1.38, 2.08)**
SO_2_	**13.35 (11.09, 15.66)**	6	**17.40 (15.01, 19.83)**
NO_2_	**7.76 (6.30, 9.23)**	6	**8.75 (7.44, 10.07)**

ER, excess risk; CI, confidence interval. [ER (95% CI)]. Bold values indicate a statistically significant difference.

In this study, a statistical model was used to analyze the effects of different air pollutants on CLRD-associated hospitalization expenses for people of different ages and sexes. The results revealed that a 10 μg/m^3^ increase in the concentrations of PM2.5 or PM10 had a significant cumulative lag effect on the CLRD hospitalization expenses for children aged 0–14 years and people aged 60 years and older. The maximum cumulative lag effects of the increase in the PM2.5 concentration on these two groups increased CLRD hospitalization expenses by 1.05% (95% CI: −0.58, 2.71%) and 1.17% (95% CI: 0.66, 1.68%), respectively, and the maximum cumulative lag effects of the increase in the PM10 concentration on these two groups increased CLRD hospitalization expenses by 2.38% (95% CI: 0.55, 4.26%) and 0.87% (95% CI: 0.32, 1.42%), respectively. When the concentrations of SO₂ and NO₂ increased by 10 μg/m^3^, they had a large cumulative lag effect on CLRD hospitalization expenses for the 15–59 age group, with hospitalization expenses increasing by 22.64% (95% CI: 17.99, 27.46%) and 7.93% (95% CI: 5.57, 10.35%), respectively.

When the PM2.5 concentration increased by 10 μg/m^3^, the cumulative lag effects increased the hospitalization expenses of CLRDs for men and women by 1.96% (95% CI: 1.29, 2.64%) and 2.19% (95% CI: 1.45, 2.94%), respectively. When the PM10 concentration increased by 10 μg/m^3^, the cumulative lag effects increased the CLRD hospitalization expenses for men and women by 1.59% (95% CI: 1.54, 1.64%) and 0.99% (95% CI: 0.43, 1.55%), respectively. When the SO₂ and NO₂ concentrations increased by 10 μg/m^3^, the cumulative lag effects for men were greater than those for women. The hospitalization expenses for men increased by 17.33% (95% CI: 13.82, 20.95%) and 7.64% (95% CI: 5.85, 9.47%), respectively. See Appendix Tables S1–S3 for details.

### 3.3 Exposure–response relationships between air pollutants and CLRD hospitalization costs

**Figure 3:**
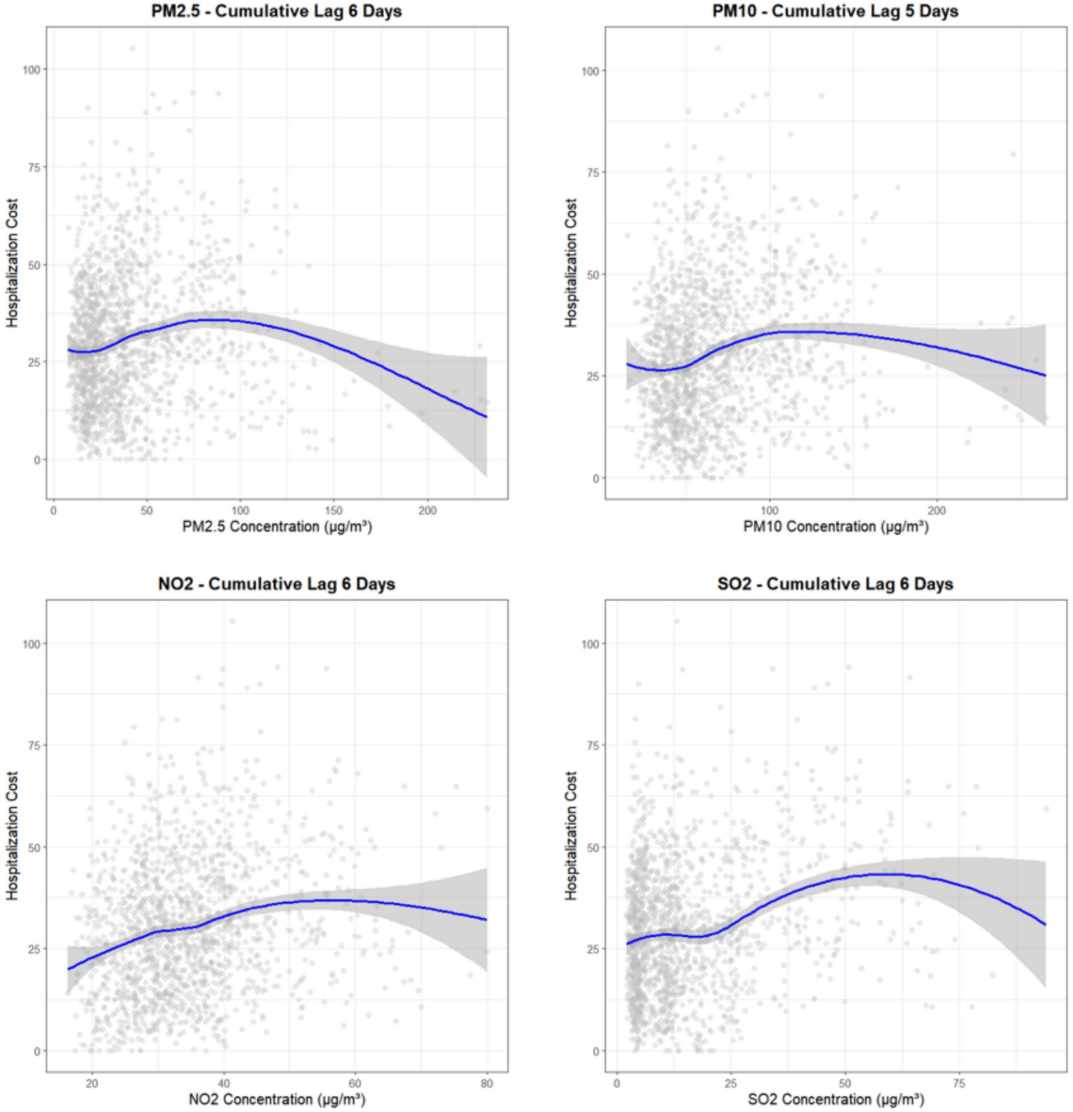
Exposure–response relationship curves between cumulative exposure to air pollutants and hospitalization costs for CLRDs.

To visually assess the dose–response relationships, we plotted scatterplots with LOESS smooth curves for each key pollutant at its peak cumulative lag period (Figure 3). The plots, which display the raw data alongside the smoothed trend and its 95% confidence interval, reveal generally monotonically increasing relationships between pollutant concentrations and hospitalization costs. This visual evidence aligns with and reinforces the findings from our quantitative GAM analysis, demonstrating a robust positive association that persists after adjusting for temporal trends, meteorological factors, and other potential confounders.

### 3.4 Association between air temperature and CLRD hospitalization costs

In Changchun city, the temperatures at the P2.5 and P97.5 percentiles were −17.91 °C and 26.81 °C, respectively. In this study, temperatures lower than −17.91 °C were defined as low temperatures, and temperatures higher than 26.81 °C were defined as high temperatures. The results of the DLNM for the association between temperature changes and CLRD hospitalization expenses in Changchun city demonstrate that after long-term trends, holiday trends, and pollutant factors were excluded, there was an association between temperature changes and hospitalization expenses for CLRDs. However, the associations between high and low temperatures and hospitalization expenses for different types of respiratory diseases differed.

**Figure 4:**
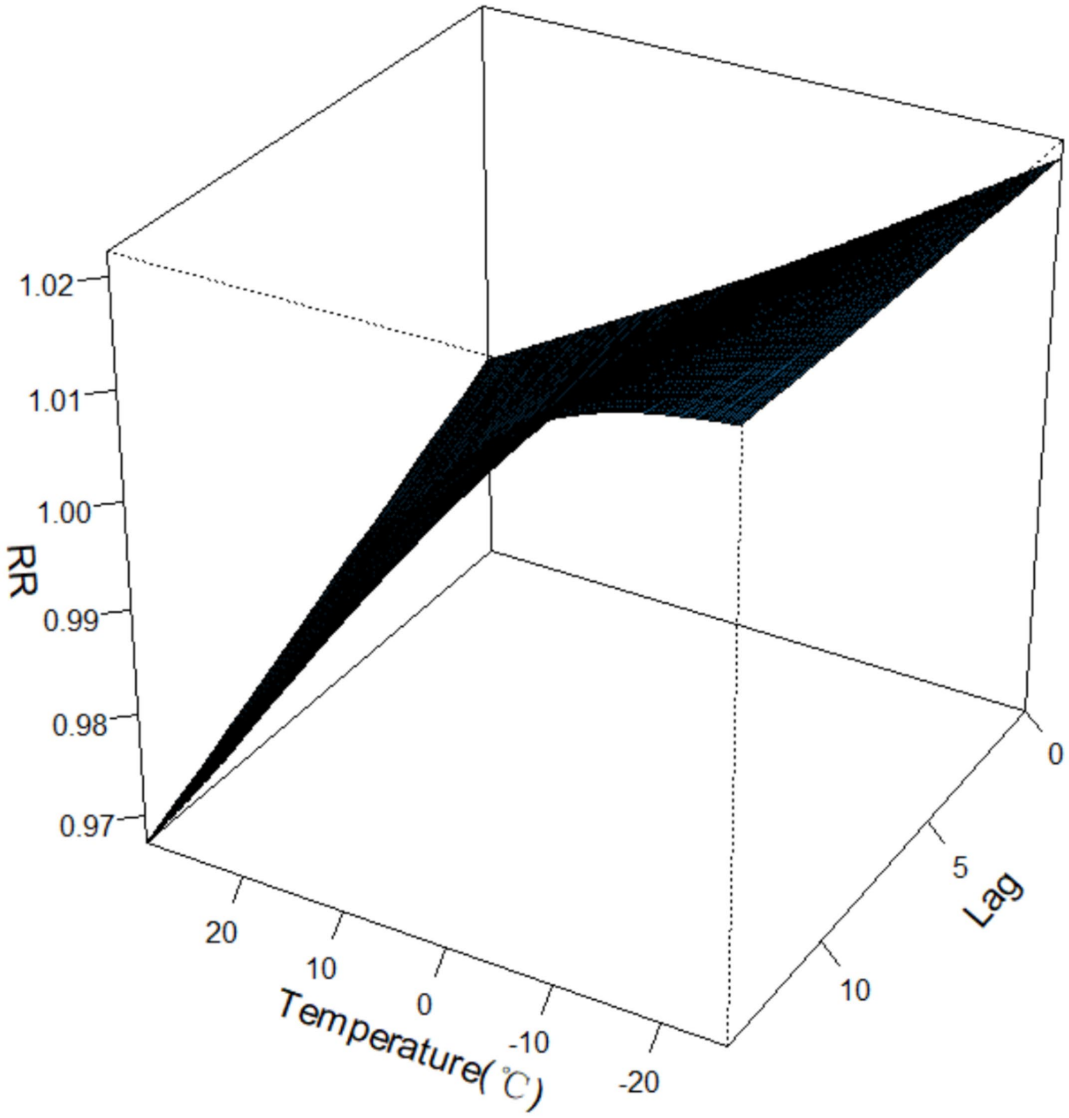
Exposure–response relationships between the CLRD costs and temperature.

The 3D diagram of the exposure–response relationship between temperature and hospitalization expenses for CLRDs demonstrated that compared with the average temperature (7.2 °C), the incidence of both low and high temperatures increased CLRD hospitalization expenses, and the relative risk (RR) of hospitalization expenses at low temperatures was greater than that at high temperatures. During the low-temperature lag period, the RR of hospitalization expenses remained relatively high, with a small variation. During the high-temperature lag period, the RR of CLRD hospitalization expenses varied widely, and the RR gradually decreased to less than 1. See Figure 4 for details.

The results of DLNM for the association between high and low temperatures and the hospitalization expenses of CLRDs demonstrated that, on low- or high-temperature days and their lagging days, the effect on CLRD hospitalization expenses for the entire population was not statistically significant. However, on low-temperature days, the hospitalization expenses of people aged 60 and older increased by 8.70% (95% CI: 1.33, 16.61%), and the ER decreased gradually and significantly from lag day 0 to lag day 5. On lag day 5, the hospitalization expenses of people aged 60 years and older increased by 3.97% (95% CI: 0.17, 7.92%). Moreover, the cumulative lag effect from lag day 0 to lag day 10 was statistically significant. From lag day 0 to lag day 10, the cumulative increase in hospitalization expenses for people aged 60 years and older was 53.53% (95% CI: 1.94, 131.22%).

In addition, at low temperatures, the ER of the hospitalization expenses of female patients with CLRDs from lag day 5 to lag day 11 gradually and significantly increased. On lag day 5 and lag day 11, the hospitalization expenses of female patients with CLRDs increased by 3.35% (95% CI: 0.35, 6.44%) and 3.92% (95% CI: 0.24, 7.73%), respectively. Moreover, the cumulative lag effect on the hospitalization expenses of female patients with CLRDs was statistically significant from lag day 9 to lag day 14. From lag day 0 to lag day 14, the cumulative increase in hospitalization expenses associated with CLRDs was 68.49% (95% CI: 20.55, 135.51%) (see Table 5). For more detailed data, see Appendix Tables S4–S9.

**Table 5:** Association and cumulative lag effect of low temperatures with CLRD hospitalization costs.

Lag days	Association effect		Cumulative lag effect	
	Aged 60 and above	Female	Aged 60 and above	Female
lag0	**8.70 (1.33, 16.61)**	2.87 (−3.46, 9.62)	**8.70 (1.33, 16.61)**	2.87 (−3.46, 9.62)
lag1	**7.74 (1.18, 14.72)**	2.97 (−2.65, 8.91)	**17.12 (2.53, 33.77)**	5.93 (−6.01, 19.38)
lag2	**6.79 (1.01, 12.89)**	3.06 (−1.85, 8.22)	**25.06 (3.59, 50.99)**	9.17 (−7.73, 29.18)
lag3	**5.84 (0.80, 11.14)**	3.16 (−1.07, 7.57)	**32.37 (4.47, 67.71)**	12.62 (−8.68, 38.90)
lag4	**4.90 (0.53, 9.47)**	3.25 (−0.33, 6.96)	**38.86 (5.15, 83.38)**	16.29 (−8.90, 48.44)
lag5	**3.97 (0.17, 7.92)**	**3.35 (0.35, 6.44)**	**44.38 (5.58, 97.44)**	20.18 (−8.42, 57.71)
lag6	3.05 (−0.30, 6.52)	**3.44 (0.90, 6.05)**	**48.79 (5.71, 109.41)**	24.32 (−7.24, 66.62)
lag7	2.14 (−0.96, 5.34)	**3.54 (1.25, 5.88)**	**51.97 (5.50, 118.92)**	28.72 (−5.40, 75.14)
lag8	1.24 (−1.82, 4.39)	**3.63 (1.33, 5.99)**	**53.85 (4.86, 125.74)**	33.40 (−2.90, 83.26)
lag9	0.34 (−2.89, 3.68)	**3.73 (1.14, 6.39)**	**54.37 (3.70, 129.80)**	**38.37 (0.22, 91.05)**
lag10	−0.55 (−4.13, 3.17)	**3.83 (0.75, 7.00)**	**53.53 (1.94, 131.22)**	**43.67 (3.90, 98.66)**
lag11	−1.43 (−5.48, 2.79)	**3.92 (0.24, 7.73)**	51.34 (−0.55, 130.29)	**49.30 (8.02, 106.36)**
lag12	−2.30 (−6.89, 2.51)	4.02 (−0.32, 8.55)	47.85 (−3.88, 127.43)	**55.30 (12.38, 114.61)**
lag13	−3.17 (−8.33, 2.29)	4.11 (−0.93, 9.41)	43.17 (−8.15, 123.18)	**61.69 (16.69, 124.03)**
lag14	−4.02 (−9.79, 2.11)	−2.93 (−7.53, 1.91)	28.31 (−5.33, 73.90)	−24.14 (−44.36, 3.43)

ER, excess risk; CI, confidence interval. [ER (95% CI)]. Bold values indicate a statistically significant difference.

### 3.5 Sensitivity analysis

To evaluate the robustness of our primary findings, we performed sensitivity analyses through two approaches. First, by adjusting the time degrees of freedom of the maximum effect lag day of the effect of air pollutants and temperature on hospitalization expenses, the GAM and DLNM of key air pollutants, temperature and hospitalization expenses were constructed. The changes in hospitalization expenses associated with CLRDs and their 95% confidence intervals were small. Second, to assess the potential confounding effects among pollutants and to investigate the independent effect of each primary pollutant, we constructed a series of two-pollutant models. Each of the four primary pollutants (PM2.5, PM10, SO_2_, and NO_2_) was entered into a model alongside one of the other three pollutants. The results demonstrated that the changes in hospitalization expenses associated with CLRDs and their 95% confidence intervals were not significant, indicating that the model was robust. In brief, the significant associations observed in the single-pollutant models remained largely robust in most two-pollutant models. While some attenuation in the effect estimates (ER%) and widening of confidence intervals occurred—a common phenomenon when correlated exposures are adjusted for—the core findings retained their statistical significance and direction. See Appendix Tables S10, S11 for details.

## 4 Discussion

From a health economics perspective, this study quantifies the direct effect of environmental exposure factors (including air pollutant concentrations and temperature changes) on the hospitalization costs of patients with CLRDs and the heterogeneity of the population. By integrating hospital case data, environmental monitoring data, and meteorological data from 2017 to 2020, combined with the GAM and DLNM, this study reveals the exposure–response relationship and its lag effect on pollutants and temperature.

Among the 24,010 patients with CLRDs, the proportion of people aged 60 years and older was 63.42%, and their hospitalization expenses accounted for 67.96% of the total, which is consistent with reports on the increasing burden of respiratory diseases under the global aging trend (38). Given their long-term environmental exposure and physiological vulnerability, the older population is at increased risk of CLRDs. Studies have demonstrated that respiratory diseases are highly prevalent among middle-aged and older population in China and are among the main causes of death among older population (39, 40). The proportion of male patients was slightly greater than that of female patients (50.83% vs. 49.17%), and the hospitalization expenses of male patients were also greater (54.22% vs. 45.78%). However, some studies have reported that women are more susceptible to respiratory diseases (41), which differs from the results of this study. Nevertheless, this study revealed that the cumulative cost risk of women under low-temperature exposure increased significantly, which may be related to the physiological differences in women (such as increased airway sensitivity) and social roles (such as the occupational environment) (42), suggesting a potential sex-specific health care utilization pattern or occupational exposure in the industrial environment. Moreover, the results of this study revealed that the social roles and work pressure of men prompt them to often relieve their emotions through smoking and that they have a greater degree of tobacco dependence. As a result of the continuous damage to the lungs caused by tobacco and other factors, the incidence and mortality of respiratory diseases in the smoking population are significantly greater than those in the nonsmoking population; thus, smoking is an important cause of respiratory diseases (43). Therefore, smoking control and occupational health education for men can help reduce the incidence of CLRDs among men and improve their health. When comparing surgical and nonsurgical patients, nonsurgical patients comprised 88.87% of the population, but surgical patients were responsible for 25.21% of the costs, indicating that severe patients consume a greater proportion of medical resources and that it is necessary to optimize the hierarchical diagnosis and treatment system to reduce the economic burden.

PM2.5 and PM10 represent particulate matter with aerodynamic diameters of less than or equal to 2.5 μm and 10 μm, respectively. Previous studies have reported that particulate matter with a particle size greater than 10 μm is prone to sedimentation and therefore can be blocked by the respiratory mucosa and cannot easily enter the lower respiratory tract, whereas particulate matter between 2.5 μm and 10 μm can enter the lower respiratory tract after being inhaled into the human body, thereby affecting gas exchange and other lung functions (28). Previous studies have also shown that the increase in the concentrations of PM2.5 and PM10 is significantly related to the number of hospitalizations for CLRDs and has obvious lag and cumulative effects (44, 45). From the perspective of health economics, this study demonstrates that for every 10 μg/m^3^ increase in the PM2.5 concentration, the hospitalization cost increases by 1.78% on the same day (lag0), and its cumulative effect reaches a peak (2.60%) after a lag of 6 days. A study in Wuhan based on the cost of illness method revealed that from 2015 to 2019, the economic losses associated with hospitalizations for respiratory diseases attributed to PM2.5 and PM10 in Wuhan were 721 million yuan and 493 million yuan, respectively. If the concentrations of PM2.5 and PM10 can reach the guideline values recommended by the World Health Organization (WHO), that is, if the annual average concentration of PM2.5 is 10 μg/m^3^ and the annual average concentration of PM10 is 20 μg/m^3^, the economic loss that can be avoided by the reduction in respiratory diseases each year could reach 194 million yuan (46). In addition, this study revealed crucial heterogeneity in the economic effects of pollutants: particulate matter (PM2.5 and PM10) had a greater influence on hospitalization costs for children (0–14 years) and older population (≥60 years). This difference in susceptibility can be attributed to a combination of physiological factors, exposure patterns, and the distinct physicochemical properties of the pollutants. The heightened effect of particulate matter on children and older population is well documented and aligns with mechanistic insights from studies on PM exposure (47, 48). Children have immature lungs, higher respiratory rates, and spend more time engaging in outdoor activities, leading to a higher intake fraction of airborne particles (48, 49). Conversely, elderly individuals suffer from declining physiological function, reduced lung clearance capacity, and a higher prevalence of preexisting cardiorespiratory conditions, increasing their vulnerability to the inflammatory and oxidative stress responses triggered by PM inhalation (47, 50). Therefore, inhalable particulate matter (PM2.5 and PM10) significantly increases the hospitalization costs associated with CLRDs, and children and elderly people are susceptible populations. In addition, the long winter in Changchun city, Jilin Province leads to a longer heating period, primarily via a coal-fired heating method. Previous studies have shown that approximately 30% of the atmospheric particulate matter in winter is generated by residential coal-fired heating (51), and more than 400,000 premature deaths are related to residential coal-fired heating in China (52). Therefore, measures such as innovating heating methods and improving the environment should be taken to reduce the generation of particulate matter continuously and enhance the protection of susceptible populations to reduce the incidence of and economic losses from respiratory disease.

SO_2_ is the most common and simplest sulfur oxide and is a common air pollutant naturally produced by geothermal activities. SO_2_ usually comes from volcanoes or is produced by anthropogenic activities, such as the combustion of coal and oil. SO_2_ dissolves in water vapor to form acid and interacts with other gasses and particles in the air to form sulfates and other inhalable particulate matter. After being inhaled into the human body, SO_2_ and its derivatives can cause oxidative damage, inflammation and apoptosis in the lungs, which can then induce mitochondrial dysfunction, leading to cell dysfunction and ultimately leading to diseases such as tracheitis, bronchial asthma and emphysema (53, 54). Nitrogen oxides are produced mainly from the combustion of fossil fuels, such as industrial and vehicle exhaust emissions. Short-term exposure of individuals to high concentrations of NO_2_ can cause respiratory tract inflammation, oxidative stress and airway hyperresponsiveness and can also damage alveolar macrophage and epithelial cell functions, thereby increasing the risk of lung infection (55, 56). Therefore, an increase in the concentrations of SO_2_ and NO_2_ increases the possibility of respiratory tract damage or aggravation of the disease in residents, leading to an increase in treatment costs. Previous studies have confirmed that exposure to SO_2_ and NO_2_ is significantly associated with the number of visits for respiratory system diseases (57, 58). The results of this study demonstrate that changes in the concentrations of SO_2_ and NO_2_ significantly affect the hospitalization costs associated with CLRDs. When the concentration of SO_2_ or NO_2_ increased by 10 μg/m^3^, the maximum single-day increase in the hospitalization costs of CLRDs reached 8.30 and 5.52%, respectively, and the maximum cumulative increase in the hospitalization costs of CLRDs within a lag of 14 days reached 17.40 and 8.75%, respectively. Therefore, the changes in the concentrations of SO_2_ and NO_2_ are strongly correlated with the increase in the hospitalization costs associated with CLRDs. Previous studies have shown that excessive SO_2_ emissions cause approximately 230,000 deaths per year, with a related economic cost of 8.179 billion yuan.

SO_2_ and NO_2_ emissions place a more substantial economic burden on the 15–59 age group. While particulate matter affects all age groups, the differential effects of these gaseous pollutants likely stem from distinct exposure pathways and physiological responses. SO_2_, a highly water-soluble gas, is primarily absorbed in the upper airways, causing immediate bronchoconstriction and irritation. NO_2_, while less soluble, can penetrate deeper into the lungs, inducing oxidative stress and enhancing airway hyperresponsiveness. The working-age population is uniquely positioned in terms of exposure: they constitute the majority of the outdoor workforce, experience significant commuting-related exposure, and are more likely to be employed in industrial or traffic-related settings where concentrations of SO_2_ (from industrial coal combustion) and NO_2_ (from vehicle emissions) are elevated (59). This occupational and commuting exposure leads to higher cumulative doses of these gasses compared with elderly people and children, who may spend more time indoors. Furthermore, the baseline lung function of healthy adults may respond to these irritant gasses with a more pronounced acute obstructive reaction—a key driver of emergency hospitalizations and associated costs—than to the chronic, progressive damage often observed with particulate matter in susceptible groups. This pattern of exposure and acute physiological response could explain why the economic burden from gaseous pollutants, reflected in hospitalization costs, is disproportionately higher for this demographic. This perspective is supported by exposure assessment studies that highlight the importance of activity patterns and microenvironments in determining personal exposure levels (50, 60). Therefore, the observed heterogeneity underscores the necessity for targeted public health interventions. Protection for children and elderly individuals should focus on reducing ambient PM levels and minimizing exposure during high-pollution days. In contrast, for the working-age population, occupational safety standards, the promotion of clean commuting options, and stricter controls on industrial and vehicular emissions of SO_2_ and NO_2_ are paramount to mitigating their distinct economic health burden.

The robustness of these findings is further supported by our sensitivity analysis using two-pollutant models, which indicated that the associations for key pollutants remained stable after mutual adjustment, lending credence to their independent effects.

After the influences of air pollutants and other meteorological factors were eliminated, low temperatures (<−17.91 °C) were significantly associated with increased hospitalization costs for CLRDs among elderly individuals aged 60 years and older and women. Research shows that the more significant health effects related to temperature may be associated with low temperatures rather than high temperatures (61). In terms of respiratory diseases, the effects of extremely low temperatures are significantly greater than those of extremely high temperatures (62), which is consistent with the results of this study. Moreover, owing to the degeneration of the physiological functions of elderly individuals, these individuals are more sensitive to temperature changes. Therefore, extremely low temperatures aggravate chronic diseases (such as cardiovascular and respiratory diseases) (61). Research has shown that women are more likely to be affected by heat waves and cold waves and to suffer from respiratory diseases (63). Changchun city is located in a cold climate region where heat waves are rare; thus, it is more affected by cold waves, which is consistent with the results of this study. The main reasons for the increased effect on females include physiological, lifestyle, and socioeconomic factors (64). Notably, the winter temperature in Changchun city is relatively low, and the period of coal-fired heating is relatively long, resulting in a large amount of pollutant emissions; moreover, pollutants do not easily diffuse in a low-temperature environment. The superposition of these low-temperature and air pollution factors leads to an increase in the incidence of CLRDs and an increase in resource consumption. Furthermore, the winter temperature is relatively low, and the influenza virus can remain active for a long time in a cold environment, which is conducive to the survival and transmission of the influenza virus, thereby increasing the risk of residents being infected with CLRDs. Therefore, it is necessary to provide health management plans for elderly individuals and women during the winter, including plans related to influenza vaccination, improvements in indoor air quality, and warmth subsidies. Moreover, the allocation of respiratory department resources should be strengthened in cold-climate cities, particularly in anticipation of low temperatures.

Our findings on the significant effect of low temperatures on hospitalization costs for elderly people and women have direct and critical implications for seasonal health policy, particularly in cold-climate cities such as Changchun. The extremely low temperatures we analyzed are a defining feature of the winter season, which is characterized not only by cold stress but also by increased emissions from heating and reduced atmospheric dispersion of pollutants. Therefore, the identified effects of low temperatures can be interpreted as capturing the core of the ‘cold season effect’ on the CLRD burden. These findings underscore the necessity of targeted winter season interventions, such as enhanced health warnings, influenza vaccination programs, and indoor air quality management for vulnerable populations.

### 4.1 Strengths and limitations

The advantages of this study include the following aspects. First, this is the first study to combine the GAM and DLNM in the context of the cold-region industrial area to quantify the economic effects of the pollutant–temperature interaction. Second, comprehensive data were obtained by integrating multisource data (hospital, meteorological, and pollutant data), controlling for time trends and confounding factors, and enhancing the reliability of the results. The third is policy orientation, which involves identifying elderly people and women as vulnerable groups and providing targets for precise cost control. However, this study also has certain limitations. First, the exposure assessment was limited. Because monitoring data from fixed sites were utilized, individual activity patterns and differences in microenvironmental exposure were not considered. Second, the influence of confounding factors, such as socioeconomic status (income and medical insurance type), on medical expenses was not included. Finally, causal inference should be drawn with caution: the observational study design cannot completely exclude residual confounding, and subsequent cohort studies are necessary for verification.

## 5 Conclusion

This study utilized Changchun city, Jilin Province, China, as a case study and systematically assessed the direct effects of environmental exposure (air pollutants and extreme temperatures) and population heterogeneity on the CLRD hospitalization costs in the context of a cold-region industrial area. The results revealed a significant association between the increase in air pollutant concentration and CLRD hospitalization costs. In particular, changes in the concentrations of SO₂ and NO₂ had a more prominent effect on the hospitalization costs of CLRDs, with the most significant effect noted for the 15–59 age group. In addition, an increase in the concentration of particulate matter (such as PM2.5 and PM10) was more strongly associated with the hospitalization costs of CLRDs for children and elderly individuals. Extreme low-temperature weather also had a significant effect on CLRD hospitalization costs for two specific groups: elderly individuals and women. This study highlights the importance of environmental exposure on the economic burden of CLRDs and accordingly puts forward a series of countermeasures and suggestions, including strengthening pollution prevention and control, implementing climate-adaptive health management strategies, and optimizing the allocation of medical resources to reduce the consumption of health resources caused by the treatment of chronic respiratory diseases.

## Data Availability

The raw data supporting the conclusions of this article will be made available by the authors, without undue reservation.

## Footnotes

1 website: http://data.cma.cn
