# Supplementary material for: Quantifying the effect of air pollution and temperature on hospitalization costs for chronic lower respiratory diseases
Source: Front Public Health. 2025 Dec 4;13:1724510. doi: 10.3389/fpubh.2025.1724510 (PMC12711853; doi:10.3389/fpubh.2025.1724510)
Supplement: Supplementary file 1 [file Table_1.docx]

Appendix Table S1 Association of air pollutant concentration changes on CLRD hospitalization costs in Different Age Groups. [ER(95%CI)]

| Lag  days | | Aged 0-14 | | | |  | Aged 15-59 | | | |  | Aged 60 and above | | | |
| --- | --- | --- | --- | --- | --- | --- | --- | --- | --- | --- | --- | --- | --- | --- | --- |
|  |  | PM_2.5_ | PM_10_ | SO_2_ | NO_2_ |  | PM_2.5_ | PM_10_ | SO_2_ | NO_2_ |  | PM_2.5_ | PM_10_ | SO_2_ | NO_2_ |
| lag0 | 1.0 (-0.6, 2.7) | 0.9 (-0.1, 1.9) | 10.5 (4.3, 17.1) | 1.0 (-3.5, 5.8) |  | 0.7 (0.1, 1.3) | 0.0 (-0.5, 0.4) | 10.1 (7.2, 13.1) | 4.3 (2.7, 5.9) |  | 0.8 (0.3, 1.2) | 0.0 (-0.2, 0.3) | 2.3 (0.3, 4.3) | 1.5 (0.5, 2.6) |  |
| lag1 | -0.8 (-2.5, 1.0) | -0.1 (-1.3, 1.0) | 4.8 (-1.2, 11.0) | -0.3 (-4.6, 4.1) |  | 0.6 (0.0, 1.2) | 0.1 (-0.4, 0.5) | 11.7 (8.8, 14.6) | 5.4 (3.8, 7.0) |  | 0.9 (0.4, 1.3) | 0.3 (0.1, 0.6) | 0.9 (-1.1, 2.9) | 1.8 (0.7, 2.8) |  |
| lag2 | -1.5 (-3.2, 0.3) | -0.1 (-1.2, 1.0) | 2.5 (-3.6, 9.0) | -4.8 (-8.9, -0.4) |  | 0.3 (-0.3, 0.9) | 0.1 (-0.3, 0.5) | 8.1 (5.3, 10.9) | 2.8 (1.3, 4.4) |  | 0.7 (0.3, 1.2) | 0.8 (0.6, 1.1) | 1.4 (-0.5, 3.4) | 1.7 (0.7, 2.7) |  |
| lag3 | 0.5 (-1.2, 2.2) | 1.5 (0.7, 2.4) | 4.3 (-2.0, 11.0) | -4.2 (-8.4, 0.1) |  | 0.5 (-0.1, 1.1) | 0.3 (-0.1, 0.7) | 11.4 (8.6, 14.3) | 3 (1.5, 4.5) |  | -0.3 (-0.7, 0.1) | 0.1 (-0.1, 0.4) | -1.2 (-3.1, 0.8) | 0.2 (-0.8, 1.2) |  |
| lag4 | 0.7 (-1, 2.4) | 1.4 (0.5, 2.3) | 5 (-1.5, 12.0) | -1.4 (-5.7, 3.0) |  | 0.0 (-0.6, 0.6) | 0.2 (-0.2, 0.6) | 9.2 (6.3, 12.1) | 2.9 (1.4, 4.4) |  | -0.9 (-1.3, -0.5) | -0.7 (-1.0, -0.4) | -3.8 (-5.7, -1.9) | -0.8 (-1.8, 0.1) |  |
| lag5 | -2.5 (-4.3, -0.6) | -0.9 (-2.1, 0.4) | 3.9 (-2.7, 11.1) | -3.9 (-8.1, 0.6) |  | 0.6 (0.0, 1.2) | -0.1 (-0.5, 0.3) | 8.8 (5.9, 11.8) | 3.9 (2.4, 5.4) |  | -0.5 (-0.9, -0.1) | 0.1 (-0.1, 0.4) | -1.9 (-3.8, 0.0) | -2.0 (-2.9, -1.0) |  |
| lag6 | -0.1 (-1.8, 1.7) | 1.2 (0.3, 2.2) | 2.1 (-4.5, 9.2) | -1.1 (-5.4, 3.4) |  | 0.4 (-0.2, 1.0) | -0.4 (-0.8, 0) | 10.3 (7.3, 13.3) | 2.4 (0.9, 3.9) |  | -0.8 (-1.3, -0.4) | -0.1 (-0.4, 0.1) | -2.3 (-4.3, -0.4) | -2.0 (-2.9, -1.0) |  |
| lag7 | 0.5 (-1.2, 2.3) | 1.2 (0.3, 2.2) | 2.9 (-3.6, 9.9) | 1.8 (-2.5, 6.4) |  | 0.4 (-0.2, 1.0) | 0.5 (0.1, 0.9) | 4.4 (1.5, 7.3) | 1.3 (-0.2, 2.8) |  | 0 (-0.4, 0.4) | -0.1 (-0.4, 0.1) | -0.2 (-2.2, 1.8) | -0.1 (-1.1, 0.9) |  |
| lag8 | 0.0 (-1.8, 1.7) | 0.2 (-0.9, 1.4) | 6.4 (-0.1, 13.3) | 1.3 (-3, 5.8) |  | -0.3 (-0.9, 0.3) | 0.1 (-0.3, 0.5) | 1.8 (-0.9, 4.6) | 2.2 (0.7, 3.7) |  | 0.7 (0.3, 1.) | 0.3 (0.0, 0.5) | 2.3 (0.3, 4.3) | 1.3 (0.3, 2.3) |  |
| lag9 | -1.7 (-3.5, 0.2) | -0.7 (-1.8, 0.5) | 5.3 (-1.0, 12.1) | -2.4 (-6.7, 2.0) |  | -0.2 (-0.8, 0.5) | 0.0 (-0.4, 0.4) | 6.2 (3.4, 9) | 2.3 (0.8, 3.8) |  | 0.7 (0.3, 1.1) | 0.8 (0.5, 1.0) | 2.6 (0.7, 4.6) | 1.4 (0.4, 2.4) |  |
| lag10 | -1.5 (-3.3, 0.3) | 0.0 (-1.1, 1.1) | -1.2 (-7.4, 5.4) | -0.4 (-4.7, 4.0) |  | -0.7 (-1.3, 0.0) | -0.6 (-1, -0.1) | 5.4 (2.6, 8.3) | 2 (0.5, 3.6) |  | -0.1 (-0.5, 0.3) | 0.1 (-0.2, 0.3) | 4.2 (2.2, 6.2) | 1.1 (0.1, 2.1) |  |
| lag11 | -0.5 (-2.2, 1.3) | -0.2 (-1.4, 1.0) | 2.0 (-4.4, 8.8) | 2.3 (-2.0, 6.8) |  | -0.2 (-0.8, 0.4) | 0.1 (-0.3, 0.5) | 6.9 (4.0, 9.8) | 3.1 (1.6, 4.6) |  | -0.2 (-0.6, 0.2) | -0.1 (-0.3, 0.2) | -2.6 (-4.5, -0.6) | 0.1 (-0.9, 1.1) |  |
| lag12 | -1.3 (-3.1, 0.5) | -0.4 (-1.7, 0.8) | 0.0 (-6.2, 6.7) | -2.4 (-6.6, 2.0) |  | -0.9 (-1.5, -0.2) | -0.5 (-0.9, -0.1) | 3.2 (0.5, 6.0) | 0.6 (-0.8, 2.2) |  | -0.3 (-0.7, 0.1) | 0.3 (0.1, 0.6) | -0.6 (-2.5, 1.3) | 0.0 (-1.0, 1.0) |  |
| lag13 | -1.5 (-3.3, 0.3) | -0.2 (-1.4, 1.0) | 2.0 (-4.4, 8.7) | -0.1 (-4.4, 4.4) |  | -0.6 (-1.2, 0.0) | -0.8 (-1.2, -0.4) | -0.6 (-3.2, 2.0) | 0.1 (-1.4, 1.7) |  | 0.0 (-0.4, 0.4) | 0.0 (-0.2, 0.3) | 0.7 (-1.2, 2.6) | 1.1 (0.1, 2.1) |  |
| lag14 | -0.5 (-2.3, 1.3) | -0.3 (-1.5, 0.9) | 5.4 (-1.1, 12.3) | 2.8 (-1.5, 7.4) |  | -0.2 (-0.8, 0.4) | -0.4 (-0.9, 0.0) | 3.6 (0.8, 6.4) | 1.4 (-0.1, 3.0) |  | 0.0 (-0.4, 0.4) | 0.1 (-0.1, 0.4) | -1.2 (-3.1, 0.8) | 0.9 (-0.1, 1.9) |  |

Note: Due to space limitations, the data in this table is rounded to one decimal place.

Appendix Table S2 Association of air pollutant concentration changes on CLRD hospitalization costs in Different Gender Groups. [ER(95%CI)]

| Lag  days | Male | | | |  | Female | | | |
| --- | --- | --- | --- | --- | --- | --- | --- | --- | --- |
|  | PM_2.5_ | PM_10_ | SO_2_ | NO_2_ |  | PM_2.5_ | PM_10_ | SO_2_ | NO_2_ |
| lag0 | 1.28 (0.82, 1.75) | 0.68 (0.36, 0.99) | 7.95 (5.85, 10.10) | 3.96 (2.77, 5.16) |  | 1.23 (0.72, 1.75) | 0.24 (-0.12, 0.60) | 4.74 (2.58, 6.94) | 2.71 (1.42, 4.02) |
| lag1 | 0.69 (0.24, 1.15) | 0.34 (0.02, 0.66) | 7.48 (5.38, 9.62) | 4.89 (3.7, 6.09) |  | 0.83 (0.34, 1.33) | 0.22 (-0.13, 0.57) | 4.05 (1.87, 6.29) | 2.98 (1.69, 4.28) |
| lag2 | 0.17 (-0.27, 0.62) | 0.07 (-0.22, 0.36) | 4.64 (2.63, 6.69) | 1.88 (0.75, 3.02) |  | 0.96 (0.48, 1.44) | 0.24 (-0.06, 0.54) | 1.71 (-0.40, 3.86) | 2.27 (1.04, 3.52) |
| lag3 | 0.62 (0.18, 1.06) | 0.46 (0.18, 0.74) | 4.43 (2.44, 6.45) | 1.70 (0.59, 2.81) |  | 1.17 (0.70, 1.64) | 0.34 (0.03, 0.64) | 5.73 (3.63, 7.86) | 3.08 (1.87, 4.30) |
| lag4 | 1.08 (0.65, 1.51) | 0.95 (0.69, 1.21) | 7.49 (5.41, 9.60) | 3.20 (2.1, 4.31) |  | 0.82 (0.34, 1.29) | 0.49 (0.19, 0.79) | 3.58 (1.49, 5.72) | 2.87 (1.68, 4.07) |
| lag5 | 1.18 (0.77, 1.6) | 0.60 (0.33, 0.87) | 6.44 (4.37, 8.55) | 3.73 (2.64, 4.83) |  | 0.79 (0.33, 1.26) | 0.42 (0.12, 0.73) | 5.63 (3.52, 7.78) | 1.80 (0.63, 3.00) |
| lag6 | 1.08 (0.64, 1.51) | 0.27 (-0.02, 0.55) | 9.25 (7.11, 11.42) | 3.59 (2.49, 4.71) |  | 0.60 (0.12, 1.08) | 0.20 (-0.11, 0.51) | 3.59 (1.48, 5.75) | 0.53 (-0.65, 1.73) |
| lag7 | 0.92 (0.49, 1.36) | 0.76 (0.46, 1.05) | 4.49 (2.40, 6.62) | 2.41 (1.31, 3.51) |  | 0.37 (-0.11, 0.85) | 0.15 (-0.18, 0.49) | 2.72 (0.59, 4.91) | 0.02 (-1.16, 1.21) |
| lag8 | -0.11 (-0.55, 0.34) | 0.39 (0.09, 0.70) | -0.27 (-2.25, 1.75) | 1.20 (0.11, 2.31) |  | -0.01 (-0.49, 0.47) | -0.04 (-0.39, 0.31) | 2.05 (-0.06, 4.21) | 0.24 (-0.94, 1.43) |
| lag9 | 0.06 (-0.38, 0.51) | 0.40 (0.13, 0.68) | 4.47 (2.46, 6.51) | 1.48 (0.38, 2.59) |  | 0.13 (-0.36, 0.61) | -0.18 (-0.50, 0.15) | 5.23 (3.11, 7.40) | 2.66 (1.45, 3.87) |
| lag10 | -0.38 (-0.82, 0.07) | 0.15 (-0.14, 0.44) | 3.74 (1.74, 5.78) | 1.55 (0.46, 2.66) |  | -0.50 (-0.99, -0.02) | -0.41 (-0.74, -0.07) | 3.26 (1.15, 5.41) | 1.29 (0.1, 2.49) |
| lag11 | -0.64 (-1.09, -0.19) | 0.15 (-0.14, 0.44) | 3.52 (1.49, 5.59) | 0.73 (-0.36, 1.82) |  | 0.06 (-0.42, 0.55) | 0.29 (-0.03, 0.60) | 1.49 (-0.62, 3.63) | 0.34 (-0.83, 1.53) |
| lag12 | -0.32 (-0.76, 0.12) | 0.03 (-0.26, 0.33) | 3.00 (1.02, 5.02) | 0.49 (-0.58, 1.57) |  | 0.03 (-0.45, 0.51) | 0.24 (-0.07, 0.55) | 0.74 (-1.35, 2.87) | 0.19 (-0.98, 1.37) |
| lag13 | -0.33 (-0.77, 0.12) | -0.18 (-0.48, 0.12) | 1.51 (-0.45, 3.5) | 0.36 (-0.73, 1.46) |  | 0.01 (-0.47, 0.50) | 0.04 (-0.28, 0.36) | 0.98 (-1.12, 3.12) | 0.73 (-0.45, 1.93) |
| lag14 | -0.02 (-0.47, 0.43) | -0.22 (-0.55, 0.10) | 2.78 (0.75, 4.85) | 1.21 (0.11, 2.33) |  | 0.23 (-0.26, 0.72) | 0.19 (-0.14, 0.53) | 2.79 (0.66, 4.97) | 1.03 (-0.17, 2.24) |

Appendix Table S3: Maximum Cumulative Lag Effects of Air Pollutant Concentrations Changes on the CLRD Hospitalization Costs in Different Populations Over a 14-Day Period. [ER(95%CI)]

| Pollutant | Age | | |  | Gender | |
| --- | --- | --- | --- | --- | --- | --- |
|  | Aged 0-14 | Aged 15-59 | Aged 60 and above |  | Male | Female |
| PM_2.5_ | 1.05 (-0.58, 2.71) | 0.70 (-0.09, 1.50) | 1.17 (0.66, 1.68) |  | 1.96 (1.29, 2.64) | 2.19 (1.45, 2.94) |
| PM_10_ | 2.38 (0.55, 4.26) | -0.02 (-0.67, 0.64) | 0.87 (0.32, 1.42) |  | 1.59 (1.05, 2.13) | 0.99 (0.43, 1.55) |
| SO_2_ | 10.52 (4.32, 17.10) | 22.64 (17.99, 27.46) | 2.41 (-0.06, 4.95) |  | 17.33 (13.82, 20.95) | 10.35 (6.85, 13.96) |
| NO_2_ | 1.01 (-3.52, 5.76) | 7.93 (5.57, 10.35) | 2.55 (1.26, 3.86) |  | 7.64 (5.85, 9.47) | 5.62 (3.81, 7.46) |

Appendix Table S4 Association and cumulative lag effect of hypothermia with CLRD hospitalization costs. [ER(95%CI)]

| Lag days | <-17.91℃ | ＞26.81℃ |
| --- | --- | --- |
| lag0 | 1.54(-3.27, 6.59) | -1.10(-6.18, 4.24) |
| lag1 | 1.58(-2.73, 6.07) | -1.23(-5.75, 3.49) |
| lag2 | 1.62(-2.20, 5.58) | -1.37(-5.33, 2.77) |
| lag3 | 1.66(-1.69, 5.12) | -1.50(-4.94, 2.07) |
| lag4 | 1.70(-1.22, 4.70) | -1.63(-4.60, 1.43) |
| lag5 | 1.74(-0.82, 4.36) | -1.76(-4.31, 0.87) |
| lag6 | 1.78(-0.50, 4.11) | -1.89(-4.14, 0.41) |
| lag7 | 1.82(-0.32, 3.99) | -2.02(-4.11, 0.12) |
| lag8 | 1.86(-0.29, 4.05) | -2.15(-4.27, 0.02) |
| lag9 | 1.90(-0.42, 4.26) | -2.28(-4.60, 0.10) |
| lag10 | 1.94(-0.67, 4.61) | -2.41(-5.07, 0.32) |
| lag11 | 1.98(-1.01, 5.05) | -2.54(-5.62, 0.65) |
| lag12 | 2.02(-1.41, 5.56) | -2.67(-6.23, 1.03) |
| lag13 | 2.06(-1.84, 6.11) | -2.80(-6.87, 1.46) |
| lag14 | 2.10(-2.30, 6.69) | -2.93(-7.53, 1.91) |

Appendix Table S5 Association and cumulative lag effect of hypothermia with CLRD hospitalization costs in Different Age Groups. [ER(95%CI)]

| Lag  days | Aged 0-14 | |  | Aged 15-59 | |  | Aged 60 and above | |
| --- | --- | --- | --- | --- | --- | --- | --- | --- |
|  | <-17.91℃ | ＞26.81℃ |  | <-17.91℃ | ＞26.81℃ |  | <-17.91℃ | ＞26.81℃ |
| lag0 | -3.64(-17.54, 12.61) | 5.27(-9.59, 22.58) |  | 4.88(-2.66, 13.00) | 2.17(-4.99, 9.88) |  | 7.74(1.18, 14.72) | -0.47(-6.62, 6.09) |
| lag1 | -3.43(-15.71, 10.64) | 2.98(-9.73, 17.48) |  | 4.37(-2.18, 11.36) | 1.49(-4.63, 8.00) |  | 6.79(1.01, 12.89) | -0.12(-5.59, 5.67) |
| lag2 | -3.23(-13.92, 8.79) | 0.74(-9.94, 12.69) |  | 3.87(-1.74, 9.79) | 0.81(-4.3, 6.19) |  | 5.84(0.80, 11.14) | 0.24(-4.58, 5.30) |
| lag3 | -3.02(-12.21, 7.13) | -1.45(-10.27, 8.23) |  | 3.37(-1.35, 8.31) | 0.13(-4.03, 4.46) |  | 4.90(0.53, 9.47) | 0.59(-3.63, 4.99) |
| lag4 | -2.81(-10.66, 5.72) | -3.60(-10.80, 4.19) |  | 2.87(-1.06, 6.95) | -0.55(-3.85, 2.87) |  | 3.97(0.17, 7.92) | 0.94(-2.76, 4.79) |
| lag5 | -2.61(-9.42, 4.72) | -5.69(-11.66, 0.68) |  | 2.37(-0.94, 5.79) | -1.21(-3.87, 1.51) |  | 3.05(-0.30, 6.52) | 1.30(-2.02, 4.73) |
| lag6 | -2.40(-8.67, 4.30) | -7.75(-13.07, -2.09) |  | 1.88(-1.09, 4.94) | -1.88(-4.24, 0.54) |  | 2.14(-0.96, 5.34) | 1.66(-1.47, 4.89) |
| lag7 | -2.19(-8.57, 4.63) | -9.75(-15.16, -4.01) |  | 1.38(-1.61, 4.47) | -2.54(-5.08, 0.08) |  | 1.24(-1.82, 4.39) | 2.02(-1.16, 5.30) |
| lag8 | -1.98(-9.09, 5.68) | -11.72(-17.8, -5.18) |  | 0.90(-2.47, 4.38) | -3.19(-6.3, 0.01) |  | 0.34(-2.89, 3.68) | 2.38(-1.08, 5.95) |
| lag9 | -1.78(-10.07, 7.28) | -13.64(-20.76, -5.88) |  | 0.41(-3.57, 4.55) | -3.84(-7.70, 0.17) |  | -0.55(-4.13, 3.17) | 2.74(-1.18, 6.81) |
| lag10 | -1.57(-11.31, 9.25) | -15.52(-23.83, -6.3) |  | -0.08(-4.80, 4.88) | -4.49(-9.19, 0.45) |  | -1.43(-5.48, 2.79) | 3.10(-1.40, 7.81) |
| lag11 | -1.36(-12.71, 11.47) | -17.36(-26.9, -6.57) |  | -0.56(-6.10, 5.31) | -5.13(-10.70, 0.79) |  | -2.30(-6.89, 2.51) | 3.46(-1.71, 8.91) |
| lag12 | -1.15(-14.18, 13.87) | -19.15(-29.91, -6.75) |  | -1.04(-7.43, 5.80) | -5.77(-12.23, 1.16) |  | -3.17(-8.33, 2.29) | 3.83(-2.07, 10.09) |
| lag13 | -0.94(-15.70, 16.41) | -20.91(-32.84, -6.86) |  | -1.52(-8.78, 6.32) | -6.40(-13.74, 1.56) |  | -4.02(-9.79, 2.11) | 4.20(-2.47, 11.32) |
| lag14 | -3.64(-17.54, 12.61) | 5.27(-9.59, 22.58) |  | 4.88(-2.66, 13.00) | 2.17(-4.99, 9.88) |  | 7.74(1.18, 14.72) | -0.47(-6.62, 6.09) |

Appendix Table S6: Association and cumulative lag effect of hypothermia with CLRD hospitalization costs in Different Gender Groups. [ER(95%CI)]

| Lag  days | Male | |  | Female | |
| --- | --- | --- | --- | --- | --- |
|  | <-17.91℃ | ＞26.81℃ |  | <-17.91℃ | ＞26.81℃ |
| lag0 | 2.51(-3.84, 9.27) | -2.11(-8.2, 4.39) |  | 2.87(-3.46, 9.62) | -2.25(-8.58, 4.52) |
| lag1 | 2.42(-3.19, 8.36) | -2.17(-7.49, 3.47) |  | 2.97(-2.65, 8.91) | -2.55(-8.07, 3.31) |
| lag2 | 2.33(-2.56, 7.48) | -2.22(-6.79, 2.57) |  | 3.06(-1.85, 8.22) | -2.84(-7.57, 2.12) |
| lag3 | 2.25(-1.95, 6.63) | -2.28(-6.11, 1.71) |  | 3.16(-1.07, 7.57) | -3.14(-7.09, 0.98) |
| lag4 | 2.16(-1.38, 5.84) | -2.33(-5.46, 0.90) |  | 3.25(-0.33, 6.96) | -3.43(-6.65, -0.11) |
| lag5 | 2.08(-0.88, 5.12) | -2.39(-4.88, 0.17) |  | 3.35(0.35, 6.44) | -3.73(-6.28, -1.11) |
| lag6 | 1.99(-0.50, 4.55) | -2.45(-4.45, -0.40) |  | 3.44(0.90, 6.05) | -4.02(-6.06, -1.93) |
| lag7 | 1.90(-0.33, 4.18) | -2.50(-4.3, -0.68) |  | 3.54(1.25, 5.88) | -4.31(-6.13, -2.45) |
| lag8 | 1.82(-0.43, 4.11) | -2.56(-4.51, -0.57) |  | 3.63(1.33, 5.99) | -4.60(-6.58, -2.58) |
| lag9 | 1.73(-0.80, 4.33) | -2.61(-5.02, -0.15) |  | 3.73(1.14, 6.39) | -4.89(-7.33, -2.39) |
| lag10 | 1.65(-1.36, 4.75) | -2.67(-5.69, 0.45) |  | 3.83(0.75, 7.00) | -5.18(-8.24, -2.02) |
| lag11 | 1.56(-2.04, 5.29) | -2.73(-6.44, 1.13) |  | 3.92(0.24, 7.73) | -5.47(-9.22, -1.56) |
| lag12 | 1.48(-2.77, 5.91) | -2.78(-7.22, 1.87) |  | 4.02(-0.32, 8.55) | -5.76(-10.24, -1.06) |
| lag13 | 1.39(-3.54, 6.58) | -2.84(-8.02, 2.64) |  | 4.11(-0.93, 9.41) | -6.04(-11.26, -0.52) |
| lag14 | 1.30(-4.33, 7.27) | -2.89(-8.83, 3.43) |  | 4.21(-1.56, 10.32) | -6.33(-12.29, 0.04) |

Appendix Table S7 Cumulative Lag Effects of High and Low Temperatures on the CLRDs Hospitalization Costs. [ER(95%CI)]

| Lag days | <-17.91℃ | ＞26.81℃ |
| --- | --- | --- |
| lag0 | 1.54(-3.27, 6.59) | -1.10(-6.18, 4.24) |
| lag0-1 | 3.14(-5.91, 13.05) | -2.33(-11.56, 7.88) |
| lag0-2 | 4.80(-7.96, 19.34) | -3.66(-16.27, 10.85) |
| lag0-4 | 8.35(-10.52, 31.19) | -6.65(-23.97, 14.62) |
| lag0-5 | 10.23(-11.10, 36.67) | -8.29(-27.11, 15.40) |
| lag0-6 | 12.19(-11.26, 41.83) | -10.02(-29.88, 15.46) |
| lag0-7 | 14.22(-11.05, 46.68) | -11.84(-32.33, 14.86) |
| lag0-8 | 16.35(-10.50, 51.24) | -13.73(-34.53, 13.67) |
| lag0-9 | 18.55(-9.67, 55.59) | -15.70(-36.54, 11.99) |
| lag0-10 | 20.85(-8.63, 59.84) | -17.73(-38.44, 9.95) |
| lag0-11 | 23.24(-7.48, 64.15) | -19.82(-40.32, 7.73) |
| lag0-12 | 25.72(-6.33, 68.75) | -21.96(-42.26, 5.49) |
| lag0-13 | 28.31(-5.33, 73.90) | -24.14(-44.36, 3.43) |
| lag0-14 | 31.00(-4.64, 79.96) | -26.36(-46.71, 1.77) |

Appendix Table S8: Cumulative Lag Effects of High and Low Temperatures on the CLRDs Hospitalization Costs in Different Age Groups. [ER(95%CI)]

| Lag  days | Aged 0-14 | |  | Aged 15-59 | |  | Aged 60 and above | |
| --- | --- | --- | --- | --- | --- | --- | --- | --- |
|  | <-17.91℃ | ＞26.81℃ |  | <-17.91℃ | ＞26.81℃ |  | <-17.91℃ | ＞26.81℃ |
| lag0 | -3.84(-19.37, 14.68) | 7.61(-9.50, 27.96) |  | 5.39(-3.16, 14.68) | 2.87(-5.37, 11.81) |  | 8.70(1.33, 16.61) | -0.82(-7.67, 6.55) |
| lag0-1 | -7.34(-33.50, 29.12) | 13.29(-18.17, 56.83) |  | 10.53(-5.73, 29.58) | 5.10(-10.08, 22.85) |  | 17.12(2.53, 33.77) | -1.28(-13.78, 13.03) |
| lag0-2 | -10.52(-43.92, 42.79) | 16.66(-26.10, 84.18) |  | 15.36(-7.77, 44.28) | 6.67(-14.24, 32.66) |  | 25.06(3.59, 50.99) | -1.40(-18.58, 19.41) |
| lag0-3 | -13.41(-51.67, 55.15) | 17.53(-33.38, 107.32) |  | 19.82(-9.32, 58.33) | 7.53(-17.89, 40.81) |  | 32.37(4.47, 67.71) | -1.16(-22.26, 25.66) |
| lag0-4 | -16.02(-57.45, 65.75) | 15.82(-40.06, 123.82) |  | 23.85(-10.44, 71.29) | 7.66(-21.11, 46.93) |  | 38.86(5.15, 83.38) | -0.58(-24.99, 31.76) |
| lag0-5 | -18.39(-61.77, 74.24) | 11.66(-46.24, 131.89) |  | 27.40(-11.17, 82.74) | 7.08(-23.97, 50.79) |  | 44.38(5.58, 97.44) | 0.36(-26.86, 37.71) |
| lag0-6 | -20.51(-64.98, 80.39) | 5.30(-51.96, 130.80) |  | 30.42(-11.56, 92.34) | 5.78(-26.51, 52.24) |  | 48.79(5.71, 109.41) | 1.66(-27.99, 43.52) |
| lag0-7 | -22.42(-67.33, 84.19) | -2.86(-57.29, 120.96) |  | 32.87(-11.66, 99.85) | 3.79(-28.81, 51.31) |  | 51.97(5.50, 118.92) | 3.35(-28.44, 49.26) |
| lag0-8 | -24.12(-69.02, 85.83) | -12.33(-62.3, 103.85) |  | 34.71(-11.54, 105.15) | 1.16(-30.93, 48.16) |  | 53.85(4.86, 125.74) | 5.43(-28.29, 55.01) |
| lag0-9 | -25.63(-70.21, 85.70) | -22.60(-67.03, 81.68) |  | 35.92(-11.31, 108.29) | -2.07(-32.98, 43.10) |  | 54.37(3.70, 129.80) | 7.94(-27.60, 60.93) |
| lag0-10 | -26.95(-71.06, 84.42) | -33.16(-71.54, 56.99) |  | 36.47(-11.10, 109.50) | -5.84(-35.08, 36.58) |  | 53.53(1.94, 131.22) | 10.90(-26.46, 67.24) |
| lag0-11 | -28.09(-71.72, 82.84) | -43.53(-75.88, 32.19) |  | 36.37(-11.1, 109.19) | -10.06(-37.39, 29.18) |  | 51.34(-0.55, 130.29) | 14.33(-24.98, 74.25) |
| lag0-12 | -29.07(-72.36, 82.02) | -53.33(-80.05, 9.18) |  | 35.61(-11.58, 107.97) | -14.68(-40.12, 21.57) |  | 47.85(-3.88, 127.43) | 18.30(-23.29, 82.41) |
| lag0-13 | -29.88(-73.17, 83.24) | -62.27(-84.04, -10.79) |  | 34.20(-12.84, 106.61) | -19.60(-43.53, 14.46) |  | 43.17(-8.15, 123.18) | 22.83(-21.55, 92.29) |
| lag0-14 | -30.54(-74.34, 88.03) | -70.16(-87.77, -27.19) |  | 32.16(-15.21, 106.01) | -24.75(-47.80, 8.49) |  | 37.41(-13.43, 118.12) | 27.98(-19.96, 104.62) |

Appendix Table 9: Cumulative Lag Effects of High and Low Temperatures on the CLRDs Hospitalization Costs in Different Gender Groups. [ER(95%CI)]

| Lag  days | Male | |  | Female | |
| --- | --- | --- | --- | --- | --- |
|  | <-17.91℃ | ＞26.81℃ |  | <-17.91℃ | ＞26.81℃ |
| lag0 | 2.51(-3.84, 9.27) | -2.11(-8.20, 4.39) |  | 2.87(-3.46, 9.62) | -2.25(-8.58, 4.52) |
| lag0-1 | 4.99(-6.91, 18.40) | -4.23(-15.08, 8.01) |  | 5.93(-6.01, 19.38) | -4.74(-15.96, 7.98) |
| lag0-2 | 7.44(-9.28, 27.24) | -6.36(-20.84, 10.78) |  | 9.17(-7.73, 29.18) | -7.45(-22.31, 10.26) |
| lag0-3 | 9.85(-11.02, 35.62) | -8.49(-25.65, 12.63) |  | 12.62(-8.68, 38.90) | -10.35(-27.79, 11.30) |
| lag0-4 | 12.23(-12.18, 43.41) | -10.62(-29.65, 13.55) |  | 16.29(-8.90, 48.44) | -13.43(-32.54, 11.08) |
| lag0-5 | 14.56(-12.79, 50.48) | -12.76(-32.96, 13.53) |  | 20.18(-8.42, 57.71) | -16.66(-36.65, 9.64) |
| lag0-6 | 16.84(-12.91, 56.74) | -14.89(-35.68, 12.6) |  | 24.32(-7.24, 66.62) | -20.01(-40.23, 7.06) |
| lag0-7 | 19.06(-12.56, 62.11) | -17.02(-37.89, 10.85) |  | 28.72(-5.40, 75.14) | -23.46(-43.37, 3.46) |
| lag0-8 | 21.22(-11.80, 66.61) | -19.15(-39.67, 8.36) |  | 33.40(-2.90, 83.26) | -26.98(-46.15, -0.98) |
| lag0-9 | 23.32(-10.68, 70.27) | -21.26(-41.12, 5.30) |  | 38.37(0.22, 91.05) | -30.55(-48.67, -6.05) |
| lag0-10 | 25.35(-9.30, 73.25) | -23.36(-42.34, 1.86) |  | 43.67(3.90, 98.66) | -34.15(-51.00, -11.51) |
| lag0-11 | 27.31(-7.79, 75.78) | -25.45(-43.46, -1.70) |  | 49.30(8.02, 106.36) | -37.75(-53.28, -17.06) |
| lag0-12 | 29.19(-6.35, 78.22) | -27.53(-44.67, -5.07) |  | 55.30(12.38, 114.61) | -41.34(-55.66, -22.40) |
| lag0-13 | 30.99(-5.25, 81.07) | -29.58(-46.19, -7.86) |  | 61.69(16.69, 124.03) | -44.88(-58.28, -27.18) |
| lag0-14 | 32.69(-4.79, 84.94) | -31.62(-48.24, -9.66) |  | 68.49(20.55, 135.51) | -48.37(-61.30, -31.12) |

Appendix Table 10: Sensitivity Analysis of Association Between the Air Pollutant Concentrations Changes and CLRDs Hospitalization Costs. [ER(95%CI)]

| **Variable** | **PM_2.5_** | **PM_10_** | **SO_2_** | **NO_2_** |
| --- | --- | --- | --- | --- |
| **Alter Degrees of Freedom** | |  |  |  |
| **df-1** | 0.88(0.58, 1.19) | 0.49(0.27, 0.71) | 7.74(6.30, 9.20) | 4.37(3.53, 5.21) |
| **df** | 1.78(1.46, 2.11) | 0.87(0.66, 1.09) | 6.84(5.40, 8.29) | 5.52(4.66, 6.38) |
| **df+1** | 1.32(0.98, 1.66) | 0.53(0.30, 0.76) | 4.68(3.24, 6.14) | 4.18(3.31, 5.06) |
| **Dual-Pollutant Model** | |  |  |  |
| **+PM_2.5_** |  | -0.04(-0.4, 0.32) | 6.23(4.68, 7.79) | 4.31(3.33, 5.30) |
| **+PM_10_** | 1.83(1.33, 2.33) |  | 7.22(5.68, 8.79) | 4.96(4.04, 5.89) |
| **+SO_2_** | 1.16(0.79, 1.53) | 0.54(0.30, 0.78) |  | 4.51(3.62, 5.41) |
| **+NO_2_** | 1.18(0.74, 1.63) | 0.47(0.21, 0.73) | 6.50(4.97, 8.05) |  |

Appendix Table 11 Sensitivity Analysis of the Association Temperature and CLRDs Hospitalization Costs - Varying Degrees of Freedom (df) for Time. [ER(95%CI)]

| **Dgrees of freedom** | **low temperatures** | **high temperature** |
| --- | --- | --- |
| df-1 | 0.52(-3.76, 5.00) | -0.47(-5.53, 4.87) |
| df | 2.10(-2.30, 6.69) | -1.10(-6.18, 4.24) |
| df+1 | 0.91(-3.4, 5.42) | -0.69(-5.79, 4.69) |
